# A New Three-Dimension Deceptive Scene Generation against Single-Pass Multibaseline InSAR Based on Multiple Transponders

**DOI:** 10.3390/s20041053

**Published:** 2020-02-15

**Authors:** Penghui Ji, Dahai Dai, Shiqi Xing, Bo Pang

**Affiliations:** State Key Laboratory of Complex Electromagnetic Environment Effects on Electronics and Information System, National University of Defense Technology, Changsha 410073, China; 19956870500@163.com (P.J.); xingshiqi_paper@163.com (S.X.); pangbo84826@126.com (B.P.)

**Keywords:** synthetic aperture radar (SAR), interferometric synthetic aperture radar (InSAR), deceptive scene, multibaseline, multiple transponders

## Abstract

The interferometry synthetic aperture radar (InSAR) deceptive jamming method utilizing two synergetic transponders can generate a false three-dimension (3D) scene in a single baseline InSAR image. However, such deceptive capability could be reduced by the multibaseline InSAR system. To obtain effective deception on multibaseline InSAR, a novel deceptive scene generation method jointly employing multiple transponders is proposed. It only demands that each transponder is modulated with a complex coefficient when generating a false point. The complex modulation coefficient can be offline calculated according to the deceptive point coordinate by solving a matrix. Besides, the complex modulation coefficient can be combined with the deceptive scene template, and thus a large 3D deceptive scene is able to be created quickly in the multibaseline InSAR image by using the fast two-dimension (2D) SAR deceptive scene generation algorithm. As long as the number of transponders is not less than the number of antennas of the multibaseline InSAR system, this proposed method is effective. The effectiveness of the proposed method is validated by computer simulations.

## 1. Introduction

As a good supplement of the light imaging, synthetic aperture radar (SAR) can acquire two-dimension (2D) high-resolution images sustainably under all-weather and all-time conditions [1]. Interferometry SAR (InSAR), a further development of SAR, employs two or more antennas to retrieve the terrain digital elevation model (DEM) of the ground surface, widely used in civil and military fields such as topographic mapping, disaster assessment, battlefield reconnaissance, strike effectiveness evaluation, etc. [2,3,4,5]. Currently, the existing InSAR systems can be divided into single baseline and multibaseline working modes [6,7]. Comparing with the traditional single baseline InSAR, the multibaseline InSAR can provide more precise retrieval height and stronger anti-jamming ability, and therefore has drawn more attentions in recent years [8]. Meanwhile, in order to protect important targets and facilities from detection and observation, the development of effective jamming methods for multibaseline InSAR should be improved in time [9].

However, the existing many traditional SAR interfering techniques such as noisy barrage jamming, shift-frequency jamming, intermittent sampling repeater jamming and scattered-wave jamming are hardly used to jam InSAR [10,11,12,13,14]. That is because the phase of the imaging output from the jamming signals is a constant related to the transponder’s position, causing the jamming signals easy to be eliminated [15,16,17]. Therefore, many researchers proposed new InSAR interfering techniques. They mainly concentrate on using a moving transponder or jointly employing two transponders [18,19,20]. In [18], a moving transponder is used to change the constant phase attached to the imaging output, and the barrage jamming is generated in the final InSAR images. However, the implementation of the moving transponder tends to be restricted by the surrounding environment. So, the authors in [19] proposed a new method jointly employing multiple transponders transmitting jamming signals in turn and realized barrage jamming against InSAR. Besides, in [20], the phase of the jamming signals was precisely controlled by modulating two transponders with different complex coefficients to produce a three-dimension (3D) deceptive scene in the InSAR image. The proposed jamming methods are all effective to some extent, but mainly appropriate for interfering single baseline InSAR. When they are applied against multibaseline InSAR, the jamming effects would decline. Especially, the deceptive jamming generated in [20] would become noisy and could not be used to produce the false scene precisely.

To generate a 3D deceptive scene in the multibaseline InSAR image, motivated by the idea of jamming multichannel SAR by using multiple transponders from [20,21,22], we proposed a novel deceptive jamming method against multibaseline InSAR systems by jointly employing multiple transponders. Without changing the process of the SAR deception jamming generated by a single transponder, each transponder only needs to be modulated with a complex coefficient to generate deception jamming in the multibaseline InSAR system. These complex coefficients can be offline calculated by solving a matrix. As long as the number of transponders is not less than the number of antennas of the multibaseline InSAR system, this proposed jamming method is effective. Besides, the rapid computational algorithm of a large two-dimension (2D) scene deceptive jamming against SAR can be utilized to generate the 3D deceptive scene, and it makes the jamming more efficient [23,24]. More specifically, the major contributions of this paper can be summarized as:

- Propose a new 3D deceptive scene generation method for jointly utilizing multiple transponders against multibaseline InSAR.
- Introduce an algorithm to acquire the complex coefficients applied to each transponder.
- Comparative experiments on the performance of the proposed jamming method for jointly employing three transponders with the existing jamming methods using two transponders against double-baseline InSAR.

The rest of the paper is organized as follows. In Section 2, we describe the process of the deceptive jamming generated by a single transponder against InSAR. Then, the reason why a single transponder and two transponders fail to encounter multibaseline InSAR is analyzed in Section 3. Section 4 presents a new method based on multiple transponders interfering multibaseline InSAR and gives an algorithm to acquire the complex coefficients applied to each transponder. In Section 5, an algorithm of fast implementation of 3D deceptive scene jamming is introduced. The simulations are presented in Section 6. Section 7 concludes this paper.

## 2. Geometrical Model and Principle for Single-Pass Multibaseline InSAR Deceptive Jamming

In this section, the geometrical model of single-pass multibaseline InSAR is first given, and then the basic principle of InSAR deceptive jamming is introduced.

### 2.1. Geometrical Model of Single-Pass Multibaseline InSAR

**Figure 1:**
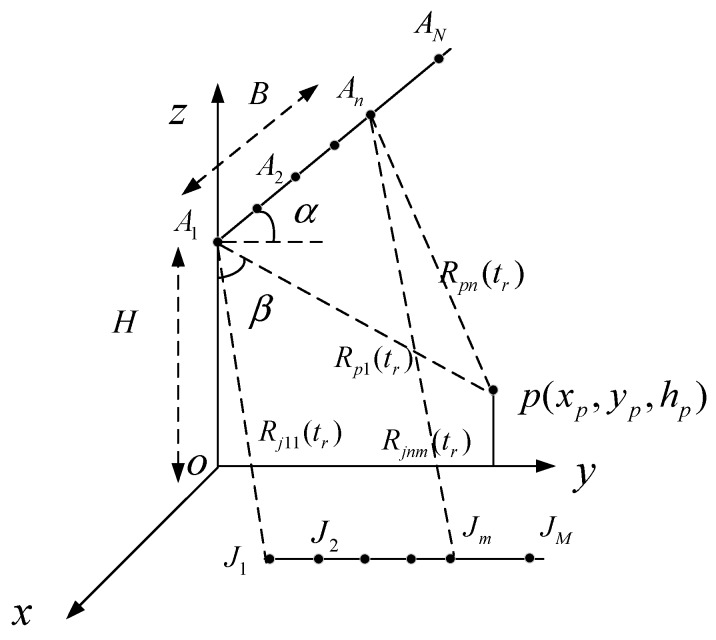
Geometry of single-pass multibaseline interferometry synthetic aperture radar (InSAR) deception jamming.

As shown in Figure 1, the multibaseline InSAR system works at the broadside mode with N receiving antennas, where the antenna $A_{1}$ serves as the transmitter and all antennas receive the echoes simultaneously. The system is mounted on an airplane, which is flying at a constant velocity $V_{a}$ parallel to the positive *x*-axis at a height of L. θ and α denote the depression angle and the inclination of the baseline, respectively. L represents the length of the baseline, which between $A_{1}$ and $A_{2}$ is $B_{1}$, and between $A_{1}$ and $A_{3}$ is $B_{2}$. Correspondingly, $B_{n-1}$ denotes as the length of the baseline between $A_{1}$ and $A_{n}$. p is a point in the three-dimension scene and its coordinate is shown by $\left(x_{p},y_{p},h_{p}\right)$. $R_{pn}(t_{a})$ denotes the instantaneous slant-range from the point p to the antenna $A_{n}$, where $t_{a}$ is the slow time. At $t_{a}=0$, the antenna $A_{1}$ lies in the y–o–z plane and its coordinate is expressed as $\left(vt_{a},0,H\right)$ varying with $t_{a}$. All M transponders are placed parallel to the *y*-axis in the x–o–y plane with the same azimuth coordinate. $R_{jnm}(t_{a})$ is the instantaneous slant-range between the antenna $A_{n}$ and the transponder $J_{m}$, where n∈(0,1,…N) and m∈(0,1,…M) serve as the index for each receiving antenna and each transponder, respectively.

From the above description, we know that the coordinate of the antenna $A_{n}$ can be expressed as $\left(vt_{a},B_{n-1}\cos(\alpha ),H+B_{n-1}\sin(\alpha )\right)$ and when n=1, $B_{0}$ is zero corresponding the length of the baseline between $A_{1}$ and $A_{1}$. Thus, the instantaneous slant-range $R_{pn}(t_{a})$ can be calculated as

(1) $$R_{pn}(t_{a})=\sqrt{{\left(vt_{a}-x_{p}\right)}^{2}+{\left(B_{n-1}\cos(\alpha )-y_{p}\right)}^{2}+{\left(H+B_{n-1}\sin(\alpha )-h_{p}\right)}^{2}}$$

Supposing that the transponder $J_{m}$ is located at $\left(x_{m},y_{m},h_{m}\right)$, then the instantaneous slant-range $R_{jnm}(t_{a})$ can be written as

(2) $$R_{jnm}(t_{a})=\sqrt{{\left(vt_{a}-x_{m}\right)}^{2}+{\left(B_{n-1}\cos(\alpha )-y_{m}\right)}^{2}+{\left(H+B_{n-1}\sin(\alpha )-h_{m}\right)}^{2}}$$

Defining $R_{Jnm}$ as the shortest distance from the antenna $A_{n}$ to the transponder $J_{m}$ then, $R_{Jnm}$ denotes as

(3) $$R_{Jnm}=\sqrt{{x_{m}}^{2}+{\left(B_{n-1}\cos(\alpha )-y_{m}\right)}^{2}+{\left(H+B_{n-1}\sin(\alpha )-h_{m}\right)}^{2}}$$

In addition, define $R_{0}$ as the distance between the scene center and the flying track.

Considering the synthetic aperture duration and the baseline are relatively short comparing with the imaging slant-range, namely $R_{Jnm},R_{0}\gg vt_{a},B_{n}$, then $R_{jnm}(t_{a})$ could be approximated as

(4) $$R_{jnm}(t_{a})\approx R_{Jnm}+\frac{{\left(vt_{a}\right)}^{2}}{2R_{0}}$$

Correspondingly, $R_{pn}(t_{a})$ could be re-calculated as

(5) $$R_{pn}(t_{a})\approx R_{p}+\frac{{\left(vt_{a}-y_{p}\right)}^{2}}{2R_{0}}$$

where

(6) $$R_{p}=\sqrt{{x_{p}}^{2}+{y_{p}}^{2}+{\left(H-h_{m}\right)}^{2}}$$

### 2.2. Principle of InSAR Deceptive Jamming

As is well known, to achieve 2D high-resolution, SAR usually emits the linear frequency-modulation (LFM) signal. Therefore, the baseband signal that the SAR transmits can be expressed as

(7) $$s_{t}(t_{r},t_{a})=w_{r}(\frac{t_{r}}{T_{p}})\exp(j\pi K{t_{r}}^{2})$$

where $w_{r}(t_{r})$ is the range window function, $t_{r}$ is the fast time, $T_{P}$ is the pulse width and K denotes the frequency modulation slope.

Therefore, the signal intercepted by the transponder $J_{m}$ can be written as

(8) $$s_{jm}(t_{r},t_{a})=s_{t}(t_{r},t_{a})\exp(j2\pi f_{0}t)⊗\delta \left(t_{r}-\frac{R_{j1m}(t_{a})}{c}\right)$$

where $f_{0}$ is the carrier frequency, ⊗ denotes the convolution operation along the fast time, δ(·) is the unit impulse response function, and c is the speed of light.

To generate a deceptive target p, the transponder demands retransmitting the intercepted signal with a time delay $\Delta \tau (t_{a})$. $\Delta \tau (t_{a})$ can be approximated as

(9) $$\Delta \tau (t_{a})\approx \frac{2\left(R_{p1}(t_{a})-R_{j11}(t_{a})\right)}{c}$$

Then, the echo from the transponder received by the antenna $A_{n}$ can be described as

(10) $$s_{jnm}(t_{r},t_{a})=s_{t}(t_{r},t_{a})\exp(j2\pi f_{0}t)⊗\delta \left(t_{r}-\Delta \tau (t_{a})-\frac{R_{j1m}(t_{a})+R_{jnm}(t_{a})}{c}\right)$$

After I/Q demodulation

(11) $$\begin{array}{ll} s_{jnm}(t_{r},t_{a}) & =s_{t}(t_{r},t_{a})⊗\delta \left(t_{r}-\Delta \tau (t_{a})-\frac{R_{j1m}(t_{a})+R_{jnm}(t_{a})}{c}\right)\\ & \times \exp\left(-j\frac{2\pi }{\lambda }\left(R_{j1m}(t_{a})+R_{jnm}(t_{a})+2\left(R_{p1}(t_{a})-R_{j11}(t_{a})\right)\right)\right) \end{array}$$

where λ is wave length of the transmitting signal and satisfies $\lambda =\frac{c}{f_{0}}$.

Last, after range Doppler (RD) imaging and image co-registration, the imaging result of the jamming signals generated by the transponder $J_{m}$ in the antenna $A_{n}$ is given as

(12) $$I_{jnm}(t_{r},t_{a})=U_{nm}G\exp(-j\frac{4\pi }{\lambda }R_{p})\exp(-j\frac{2\pi }{\lambda }R_{Jnm})$$

(13) $$G=\sin c[B_{r}(t_{r}-\frac{2R_{p}}{c})]\sin c[B_{a}(t_{r}-\frac{x_{p}}{V_{a}})]$$

where G is the envelop function of the imaging result of the false target p, $U_{nm}$ is the complex amplitude of the deceptive false target p created by the transponder $J_{m}$ in the SAR image of antenna $A_{n}$, $B_{r}$ is the frequency band of range, and $B_{a}$ is the frequency band of the azimuth. Assuming that all transponders are completely the same, then $U_{nm}$ can be seen as a constant. Without loss of generality, assume $U_{nm}=U_{0}$, and then the imaging result becomes

(14) $$I_{jnm}(t_{r},t_{a})=U_{0}G\exp(-j\frac{4\pi }{\lambda }R_{p})\exp\left(-j\frac{2\pi }{\lambda }(R_{J1m}+R_{Jnm})\right)$$

Equation (14) reveals that the phase of the final deceptive jamming result is only related to the shortest length $R_{p}$ from the target p to the flight path and the shortest distance $R_{Jnm}$ from the transponder $J_{m}$ to the antenna $A_{n}$.

## 3. Analysis of the Traditional Deceptive Jamming Method against Single Baseline InSAR

This section first analyzed the limitation of the traditional deceptive jamming generated by a single transponder and then introduced an effective jamming method for jointly employing two transponders against the single baseline InSAR. In the end, the reason why double transponders fail to deceive multibaseline InSAR was given.

### 3.1. The Limitation of a Single Transponder

From the above analysis, we learnt that the final phase of the imaging result for the antenna $A_{n}$, which decides the retrieval height of the false target, only depends on $R_{p}$ and $R_{Jnm}$. In the following, to better analyze the effects on the SAR image resulted from the phase of the imaging output of the jamming signals, we only considered that jamming was generated by a single transponder against single baseline InSAR. Without loss of generality, assume n∈(1,2), m=1. Then, the deceptive false target in the SAR image for antenna $A_{1}$ can be obtained as

(15) $$I_{j11}(t_{r},t_{a})=U_{0}G\exp(-j\frac{4\pi }{\lambda }R_{p})\exp(-j\frac{4\pi }{\lambda }R_{J11})$$

Similarity, the imaging output of the false target in the image domain for antenna $A_{2}$ can be written as

(16) $$I_{j21}(t_{r},t_{a})=U_{0}G\exp(-j\frac{4\pi }{\lambda }R_{p})\exp\left(-j\frac{2\pi }{\lambda }(R_{J11}+R_{J21})\right)$$

Accordingly, the phase difference of the false target p between the two SAR images is derived as

(17) $$\hat{\phi }=\arg(I_{j11}{I_{j21}}^{*})=-\frac{2\pi }{\lambda }(R_{J11}-R_{J21})$$

Obviously, the phase difference ϕ in Equation (17) is a constant and correspondingly the retrieval height is also a constant, which does not change with the setting different height of the false target. Moreover, the false target even can be eliminated by compensating the second antenna with the phase difference. Therefore, the traditional deceptive jamming generated by a single transponder plays a limit part in the InSAR systems.

### 3.2. Synergy Jamming with Two Transponders against Single Baseline InSAR

In Section 3.1, we showed a single transponder could not create deceptive jamming in single baseline InSAR due to the constant phase difference between two images of two receiving antennas. However, if jointly using two transponders, although the phase difference between two images of two receiving antennas is still a constant for each transponder, an expected synthetic phase difference can be obtained by modulating each transponder intercepting signal with a complex coefficient. Therefore, a false target with the setting height can be generated.

Considering the azimuth position of the transponder has no difference in the final phase of the false target in the image domain, for simplification, two transponders $J_{1}$ and $J_{2}$ are located at the same azimuth position along the ground-range direction. The corresponding complex modulation coefficients modulated in two transponders denote as $Q_{1}$ and $Q_{2}$, respectively. Then the synthetic jamming signals of the two transponders for the antenna $A_{1}$ and $A_{2}$ in the image domain can be respectively written as

(18) $$\begin{array}{l} I_{j112}(t_{r},t_{a})=I_{j11}(t_{r},t_{a})+I_{j12}(t_{r},t_{a})\\ =U_{0}G\exp(-j\frac{4\pi }{\lambda }R_{p})\left(Q_{1}\exp(-j\frac{4\pi }{\lambda }R_{J11})+Q_{2}\exp(-j\frac{4\pi }{\lambda }R_{J12})\right) \end{array}$$

(19) $$\begin{array}{l} I_{j212}(t_{r},t_{a})=I_{j21}(t_{r},t_{a})+I_{j22}(t_{r},t_{a})\\ =U_{0}G\exp(-j\frac{4\pi }{\lambda }R_{p})\left\{Q_{1}\exp\left(-j\frac{2\pi }{\lambda }(R_{J11}+R_{J21})\right)+Q_{2}\left(-j\frac{2\pi }{\lambda }(R_{J12}+R_{J22})\right)\right\} \end{array}$$

Thus, to obtain the desired deceptive jamming target, the phase difference between $I_{j112}(t_{r},t_{a})$ and $I_{j212}(t_{r},t_{a})$ should satisfy

(20) $$\hat{\phi }=\arg(I_{j112}{I_{j212}}^{*})=\Delta \varphi (x_{p},y_{p})$$

where $\Delta \varphi (x_{p},y_{p})$ is the desired phase of the false target p.

Obviously, there are arrays of solutions $Q_{1}$ and $Q_{2}$ that satisfy the phase difference $\Delta \varphi (x_{p},y_{p})$. Without loss of generality, we assumed that a set of solutions $Q_{1}$ and $Q_{2}$ satisfied $\left(Q_{1}\exp(-j\frac{4\pi }{\lambda }R_{J11})+Q_{2}\exp(-j\frac{4\pi }{\lambda }R_{J12})\right)=\exp(0)$, and then combined (18), (19) and (20), rewriting them in the formation of matrix as AQ=b

(21) $$\left[\begin{array}{cc} a_{11} & a_{12}\\ a_{21} & a_{22} \end{array}\right]\left[\begin{array}{c} Q_{1}\\ Q_{2} \end{array}\right]=\left[\begin{array}{c} 1\\ b_{2} \end{array}\right]$$

where $a_{11}=\exp(-j\frac{4\pi }{\lambda }R_{J11})$, $a_{12}=\exp(-j\frac{4\pi }{\lambda }R_{J12})$, $a_{21}=\exp\left(-j\frac{2\pi }{\lambda }(R_{J11}+R_{J21})\right)$, $a_{22}=\exp\left(-j\frac{2\pi }{\lambda }(R_{J12}+R_{J22})\right)$ and $b_{2}=\exp\left(\Delta \varphi (x_{p},y_{p})\right)$.

Solving Equation (21), the complex modulation coefficients $Q_{1}$ and $Q_{2}$ can be obtained. By applying the two complex modulation coefficients in two transponders, a setting height can be realized in the setting point $\left(x_{p},y_{p}\right)$.

### 3.3. The Limitation of Two Transponders against Multibaseline InSAR

In Section 3.2, an effective deceptive jamming method with two synergetic transponders against single baseline InSAR was introduced. However, the jamming performance could be reduced when the two synergetic transponders are utilized to interfere multibaseline InSAR. Since the phases of the synthetic jamming signals could not satisfy the phases corresponding to the false targets’ heights in other antennas except the antenna $A_{1}$ and $A_{2}$. For example, the InSAR system possesses two baselines, and namely there are three antennas. Then, applying the two complex coefficients to the 3rd transponder $J_{3}$, the output of the imaging result in antenna $A_{1}$ shown by(18), antenna $A_{2}$ shown by (19) and in antenna $A_{3}$ is written as

(22) $$\begin{array}{l} I_{j312}(t_{r},t_{a})=I_{j31}(t_{r},t_{a})+I_{j32}(t_{r},t_{a})\\ =AG\exp(-j\frac{4\pi }{\lambda }R_{p})\left\{Q_{1}\exp(-j\frac{4\pi }{\lambda }R_{J31})+Q_{2}\exp\left(-j\frac{2\pi }{\lambda }(R_{J31}+R_{J32})\right)\right\} \end{array}$$

Apparently, the phase difference of the false target between $I_{j112}(t_{r},t_{a})$ and $I_{j312}(t_{r},t_{a})$ does not satisfy (20). Therefore, the deceptive jamming generated by two synergetic transponders plays a limit role against the multibaseline InSAR.

## 4. The Proposed Method

Based on the previous analysis, we could conclude that to obtain the desired phase satisfying the height of the deceptive target, the number of transponders should not be less than the number of antennas of the multibaseline InSAR system. Thus, in this section, we proposed a new deceptive jamming method against multibaseline InSAR based on multiple transponders.

Reconsidering Equation (14), it gives the imaging result of the false target p generated by the transponder $J_{m}$ in the antenna $A_{n}$. Assume there are *M* transponders working synergistically, and to generate the false target p, each transponder is modulated with a complex modulation coefficient when generating the jamming signal. Then the composite jamming signals of all transponders for the antenna $A_{n}$ in the image domain can be expressed as

(23) $$I_{n}^{J}(t_{r},t_{a})=UG\exp(-j\frac{4\pi }{\lambda }R_{p})\sum_{m=1}^{M}Q_{m}\exp\left(-j\frac{2\pi }{\lambda }(R_{J1m}+R_{Jnm})\right)$$

where n∈{1,2…N}, m∈{1,2…M}, N≤M and $Q_{m}$ denotes the complex modulation coefficient modulated in the transponder $J_{m}$.

Accordingly, if the phase difference of the imaging output of the jamming signals between two co-registration images for the antenna $A_{n}$ and $A_{1}$ denotes as

(24) $$\phi_{n}=\arg(I_{1}^{J}{I_{n}^{J}}^{*})=\Delta \varphi_{n}(x_{p},y_{p})$$

and assuming $\sum_{m=1}^{M}Q_{m}\exp\left(-j\frac{2\pi }{\lambda }(R_{J1m}+R_{J1m})\right)=\exp(0)$, then, the summing term in (23) should satisfy

(25) $$\sum_{m=1}^{M}Q_{m}\exp\left(-j\frac{2\pi }{\lambda }(R_{J1m}+R_{Jnm})\right)=\exp(\phi_{n})$$

To obtain the desired height of the false target, the Equation (24) should satisfy the phase corresponding to the real target’s height for all N antennas. That is to say, if there is an ambiguous phase caused by the baseline $B_{n}$, it should be added to the corresponding phase difference $\Delta \varphi_{n}(x_{p},y_{p})$.

Rewrite Equation (25) in matrix as AQ=ϕ.

(26) $$\left[\begin{array}{ccccc} a_{11} & a_{12} & \ldots & \ldots & a_{1M}\\ a_{21} & a_{22} & \ldots & \ldots & a_{2M}\\ \vdots & \vdots & \ddots & \vdots & \vdots \\ \begin{array}{c} \vdots \\ \vdots \end{array} & \begin{array}{c} \vdots \\ \vdots \end{array} & \begin{array}{c} \ldots \\ \ldots \end{array} & \begin{array}{c} a_{nm}\\ \ldots \end{array} & \begin{array}{c} \vdots \\ \vdots \end{array}\\ a_{N1} & a_{N2} & \ldots & \ldots & a_{NM} \end{array}\right]\left[\begin{array}{c} Q_{1}\\ Q_{2}\\ \vdots \\ \begin{array}{c} Q_{n}\\ \vdots \end{array}\\ Q_{N} \end{array}\right]=\left[\begin{array}{c} \exp(\phi_{1})\\ \exp(\phi_{2})\\ \vdots \\ \begin{array}{c} \exp(\phi_{n})\\ \vdots \end{array}\\ \exp(\phi_{N}) \end{array}\right]$$

where $a_{nm}=\exp\left[j\frac{2\pi }{\lambda }(R_{Jnm}+R_{J1m})\right]$, and $\phi_{n}$ is shown in (24).

By solving Equation (26), the complex modulation coefficients can be obtained. By applying these complex modulation coefficients in the transponders, the desired height of the false target can be obtained.

Besides, in practical electronic war (EW), the jamming systems perform better when the number of the transponders is more than the number of antennas of InSAR systems [25,26]. There are two reasons for that. On the one hand, more transponders mean lower transmitting power for each transponder and thus, the possibility of the transponder being detected is reduced. On the other hand, once a transponder breaks down, other transponders can continue generating deceptive jamming after adjusting the complex coefficients modulated in them, thus, the robustness of the jamming system is also stronger.

## 5. The Fast Implementation of 3D Deceptive Scene Jamming

From the above analysis, we know that the new deceptive jamming method does not require the transponder to change too much compared to the traditional SAR deceptive jamming method and only to be modulated with a complex modulation coefficient when generating the jamming signals of a deceptive target. We could also find that the complex modulation coefficient could be equivalent to the reference complex scattering coefficient of the false target. Thus, we could combine the complex modulation coefficient with the reference complex scattering coefficient of the false target to form a new scattering coefficient. In this way, the jamming system using the proposed jamming method to generate a 3D deceptive scene is just like using the traditional SAR deception jamming method to generate a 2D deceptive scene.

Assume that the deceptive scene template is a K×L matrix, and K denotes the number of false point targets in azimuth direction and L denotes the number of false point targets in the ground range direction. Besides, denote h(k,l) and σ(k,l) as the DEM template and the reference complex scattering template, respectively. According to the geometric and signal parameters of InSAR system provided by electronic reconnaissance system, the complex coefficients modulated in the transponder $J_{m}$ can be offline calculated as $Q_{m}(k,l)$. Then, the new complex scattering coefficients of the deceptive scene template for the transponder $J_{m}$ becomes

(27) $$\gamma_{m}(k,l)=Q_{m}(k,l)*\sigma (k,l)$$

To generate a 3D deceptive scene, the existing fast deceptive jamming method against SAR can be used. Here, we used the algorithm proposed in [23]. In this fast algorithm, the modulated deceptive template of each transponder is decomposed into the slow-time-dependent and the slow-time-independent terms in the range frequency-azimuth time domain. The slow-time-independent terms can be calculated offline and the slow-time-dependent terms can also be realized rapidly by the fast Fourier transform (FFT), which greatly reduces the computational burden.

## 6. Simulation Experiments

In this section, to verify the effectiveness of the proposed method, simulations were provided based on a double-baseline InSAR system with three receiving antennas $A_{1}$, $A_{2}$ and $A_{3}$ shown as in Figure 1. The InSAR system parameters are listed in Table 1 where the length of the baseline between antenna $A_{1}$ and $A_{2}$ was 2 m, and between antenna $A_{1}$ and $A_{3}$ it was 5 m.

The program of the simulation experiments was written with the Matlab language and carried out in the Matlab 2016 environment. In the simulation, first, we simulated the jamming signals based on the proposed method and planted them in the background signals; and then, used the range-Doppler (RD) algorithm to obtain the SAR image of each antenna and operate image coregistration; next, after image coregistration, we extracted the phase interferogram corresponding to the baseline and carried out the phase unwrapping operation. Last, according to the absolute interferogram after unwrapped, we obtained the height of the false target. Besides, the jamming signal power to clutter signal power ratio (JCR) was 5 dB in the simulation experiments.

**Figure 2:**
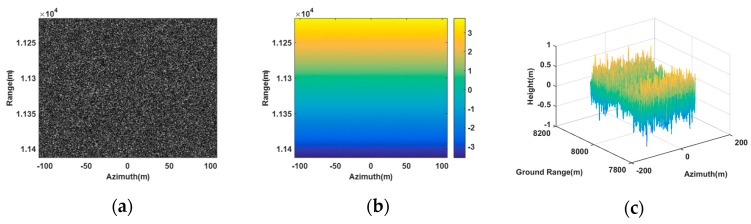
The real scene. (**a**) SAR image in the antenna $A_{1}$. (**b**) Absolute interferogram after unwrapped. (**c**) Digital elevation model (DEM) of the real scene.

Figure 2 shows the image scene that the false point target and the deceptive scene were planted in, and it was a flat plane with an area of 200 m × 200 m in the ground range and azimuth directions. 

### 6.1. False Point Target Simulation

In this section, we verified the effectiveness of the proposed method based on whether the coordinates of the generated false targets match the set values. Therefore, we set five false point targets in the image scene, denoting as $p_{1}$, $p_{2}$, $p_{3}$, $p_{4}$ and $p_{5}$, respectively, and their coordinates are listed in Table 2. Among all false point targets, $p_{1}$, $p_{2}$ and $p_{3}$ were located at the same azimuth coordinate 0 m, while $p_{3}$, $p_{4}$ and $p_{5}$ shared the same ground range 8000 m. To generate the jamming signals, three transponders $J_{1}$, $J_{2}$ and $J_{3}$ were utilized, being located at (0,8000,0), (0,8020,0) and (0,7980,0), respectively. Based on the proposed method, the complex modulation coefficients modulated in each transponder for all false targets should be first accurately calculated according to Equation (26). As shown in Table 3, $Q_{1}$, $Q_{2}$ and $Q_{3}$ were denoted as the complex modulation coefficient corresponding to the transponder $J_{1}$, $J_{2}$ and $J_{3}$, respectively. To better verify the effectiveness of the proposed method, a comparative experiment utilizing two transponders $J_{1}$ and $J_{2}$ was also carried out.

**Figure 3:**
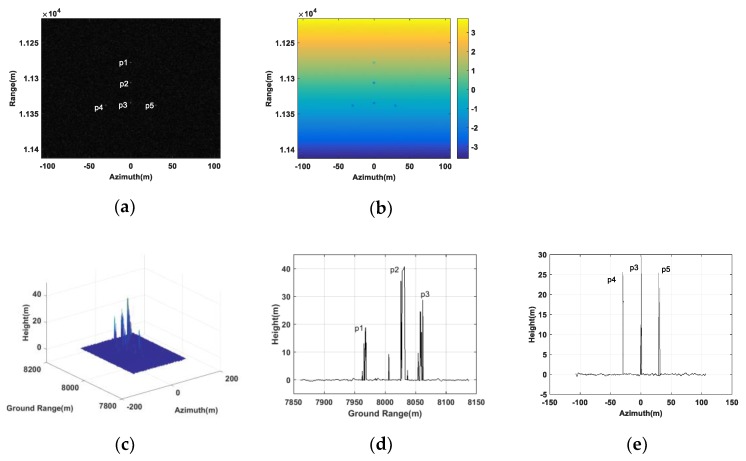
False point targets simulation based on three transponders. (**a**) SAR image in the antenna $A_{1}$. (**b**) Absolute interferogram after unwrapped. (**c**) DEM of the false targets. (**d**) A slice graph of DEM of the false targets in ground range. (**e**) A slice graph of DEM of the false targets in azimuth.

Figure 3 gives the results for interfering the double-baseline InSAR system with three transponders. In Figure 3a, five false targets are indicated in the SAR image of the antenna $A_{1}$. The corresponding absolute interferogram after being unwrapped is shown in Figure 3b. Figure 3b revealed that, due to the different heights between the false targets and the real scene, the absolute interferometric phases of the false targets differed from their surrounding scene. Figure 3c shows the retrieval height of the false point targets. From Figure 3c, we could see five peaks corresponding to the five false targets. Figure 3d,e gives the slice graphs of Figure 3c in the ground range and azimuth directions, respectively. From Figure 3d,e, it is obvious that the retrieval height of the five point targeted all tallies with their setting values. Table 4 gives the quantitative estimated coordinates, and from it we can see that the quantitative estimated coordinates were almost the same as the setting with only little errors. This indicates that the proposed method worked better in false point targets generation.

**Figure 4:**
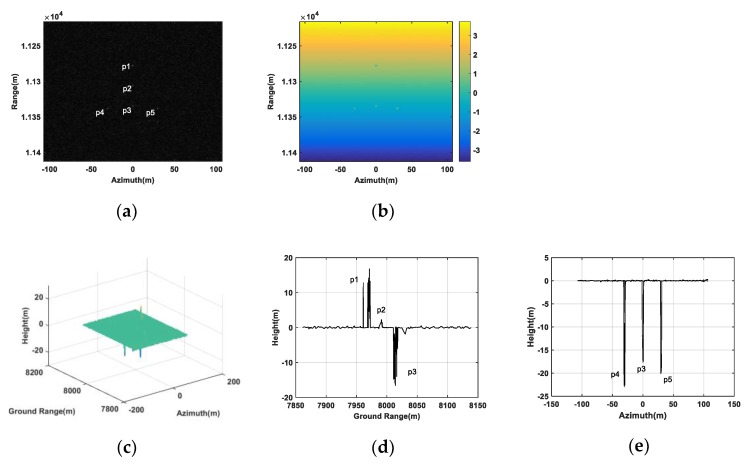
False point targets simulation based on two transponders. (**a**) SAR image in the antenna $A_{1}$. (**b**) Absolute interferogram after unwrapped. (**c**) DEM of the false targets. (**d**) A slice graph of DEM of the false targets in ground range. (**e**) A slice graph of DEM of the false targets in azimuth.

However, when only two transponders were utilized to generate jamming, the created false point targets shown in Figure 4 differed greatly from the setting. In Figure 4a, all five false targets still can be seen in the image of the antenna $A_{1}$. However, in Figure 4b, the absolute interferogram after being unwrapped, we can see the interferometric phases of the false targets were not clearly shown, where the interferometric phase of the point target $p_{3}$ even mixed with the surrounding scene. In Figure 4c, the retrieval heights of some false targets were below zero and obviously disobeyed the setting values where all heights were positive. Besides, Figure 4d,e describes, due to the inaccurate retrieval heights, these false targets were not mapped at their setting positions, after being transformed from a slant range to ground range. Therefore, it could be concluded that two transponders could not be used to generate deceptive jamming with high fidelity against double-baseline InSAR.

Based on above analysis, we could also conclude that the number of transponders should not be less than the number of antennas of multibaseline InSAR systems when the transponders were utilized to generate deceptive jamming.

### 6.2. 3D Deceptive Scene Simulation

**Figure 5:**
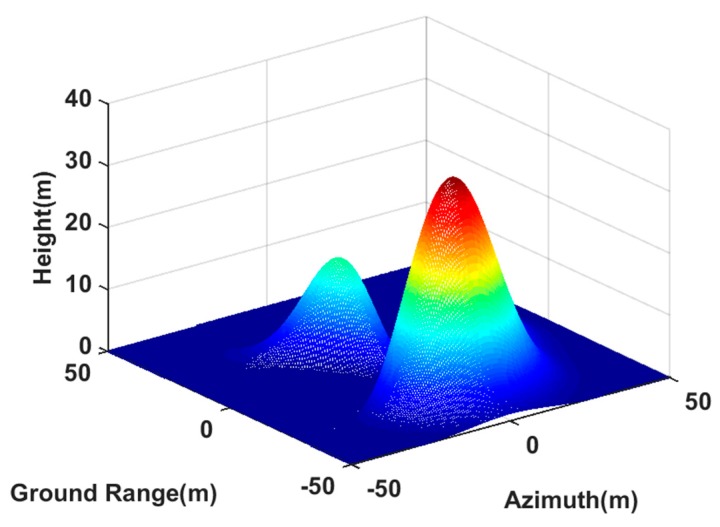
The 3D deceptive scene.

In this section, a 3D deceptive scene was generated based on our proposed method. The 3D deceptive scene template was created using peaks function in Matlab, and it was an area of 100 m × 100 m in the ground range and azimuth directions. The elevation map is shown in Figure 5, where the maximum height was 35 m. The center of the deceptive scene is located at the center of image scene. The reference scattering coefficient was modeled as Gaussian white noise. Besides, the arrangement of the transponders and the InSAR systems parameters were the same as Section 6.1. 

**Figure 6:**
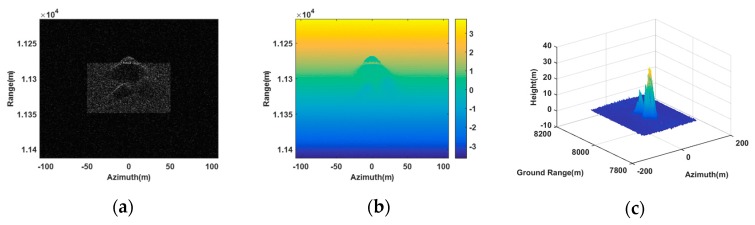
3D deceptive scene generation simulation based on three transponders. (**a**) SAR image in the antenna $A_{1}$. (**b**) Absolute interferogram after unwrapped. (**c**) DEM of the false scene.

Figure 6 is the imaging result of our proposed method using three synergetic transponders. From the SAR image of the antenna $A_{1}$, as shown in Figure 6a, it can be seen that the generated scene was similar to the deceptive scene. The retrieval height of the deceptive scene shown in Figure 6c was also consistent with the deceptive template (as shown by Figure 5), where the maximum height was 34.63 m approaching the setting maximum value. Thus, our proposed method was valid in 3D deceptive scene generation. 

**Figure 7:**
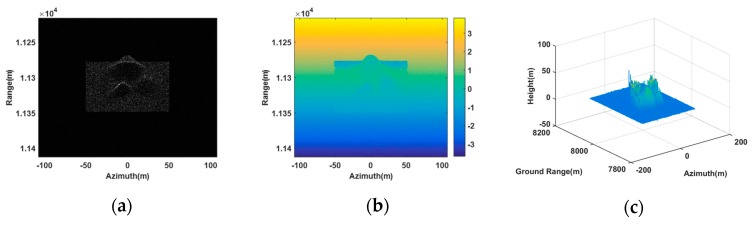
3D deceptive scene generation simulation based on two transponders. (**a**) SAR image in the antenna $A_{1}$. (**b**) Absolute interferogram after unwrapped. (**c**) DEM of the false scene.

Figure 7 shows the imaging result of the method in [20] using two synergetic transponders. In Figure 7c, the DEM was greatly different from the deceptive template, because the phase of the composite jamming signal generated by two transponders was messy in the 3rd receiving antenna. Moreover, the maximum height in Figure 7c was 65.46 m, which did not tally with the setting maximum value. This also confirmed the limitation of dual transponders in jamming multibaseline InSAR.

To quantitatively verify the effectiveness of the proposed method in generating the 3D deceptive scene, we chose three points $q_{1}$, $q_{2}$ and $q_{3}$ in Figure 5 to compare their retrieval heights with their setting heights. The coordinates of three points $q_{1}$, $q_{2}$ and $q_{3}$ in the image scene are (0, 7973, 35), (−15, −7962, 6.94) and (10, 7981, 14.67), respectively. The retrieval coordinates near the setting coordinates are shown in Table 5. It is shown that the jamming based on three transponders worked well, and the retrieval heights were all at the acceptable level. However, the jamming generated by two transponders played a limit role, which had a large height estimation error. Thus, it could be found that the proposed method was effective to generate the 3D deceptive scene.

The simulation results verified the effectiveness of the proposed method. In fact, the applicability of the proposed method was also feasible in practice. First, with the rapid development of the electronic reconnaissance techniques [27,28], the InSAR system parameters required for generating the jamming signals could be all accurately estimated. Besides, due to using the fast algorithm, the computational load was dramatically reduced. Last, according to the simulations, the transponders were relatively close to each other and could work synergistically by wired means. Therefore, in the short interference time, the transponder was able to generate the jamming signals effectively and timely.

## 7. Conclusions

In this paper, a novel 3D deceptive scene generation method based on multiple transponders for interfering multibaseline InSAR was proposed, where the number of transponders should not be less than the number of antennas of the multibaseline InSAR systems. It derived from the traditional SAR deceptive jamming and only required each transponder to be modulated with the complex coefficients related to the deceptive scene template when generating the jamming signals. By applying these coefficients in each transponder, the phase of the composite jamming signals in each InSAR antenna satisfied the desired DEM. Moreover, this method was also computationally efficient for a large 3D deceptive scene generation, because these coefficients could be calculated offline and combined with the reference complex scattering coefficient of the deceptive template. Therefore, by using the existing SAR 2D deceptive jamming fast algorithms, the 3D deceptive scene could be created rapidly. This makes the proposed jamming method suitable for EW environments that require high real-time conditions. Simulation results show that the proposed method could effectively generate 3D deceptive scenes in multibaseline InSAR image.

Besides, in this paper, we only gave the method for generating the jamming signals against the single polarimetric InSAR. However, the proposed method is also appropriate for jamming the fully polarimetric InSAR. As is well known, the fully polarimetric InSAR increases the observation information on target scattering characteristics and can help to provide more physical features of the target. While using our proposed method to generate the jamming signals, the scattering coefficients of the false targets are fully utilized. Therefore, for the fully polarimetric InSAR, by performing polarization modulation for the jamming signals, the synergetic transponders are able to generate the false targets with high fidelity.

## Figures and Tables

**Table 1:** Parameters of double-baseline InSAR.

Parameters	Value
Carrier frequency	10 GHz
Platform altitude	8000 m
Platform velocity	100 m/s
Baseline length	2 m, 5 m
Looking angle	45°
Baseline angle	45°
Range resolution	0.5 m
Azimuth resolution	0.5 m

**Table 2:** The coordinates of the false point target.

Targets	$p_{1}$	$p_{2}$	$p_{3}$	$p_{4}$	$p_{5}$
Coordinates	(0,7970,20)	(0,8030,40)	(0,8060,30)	(−30,8060,25)	(30,8060,25)

**Table 3:** Complex coefficients of different false targets corresponding to three transponders.

Targets	$Q_{1}$	$Q_{2}$	$Q_{3}$
$p_{1}$	0.2996 + 0.6792i	0.1356 − 0.0133i	0.3637 + 0.1514i
$p_{2}$	4.4404 + 7.2291i	4.6949 − 1.4513i	−4.4356 − 1.1702i
$p_{3}$	6.2796 + 3.0655i	3.0871 − 3.4188i	−3.2170 + 1.0517i
$p_{4}$	5.2715 + 6.3771i	4.4824 − 2.0585i	−4.3243 − 0.4994i
$p_{5}$	5.2715 + 6.3771i	4.4824 − 2.0585i	−4.3243 − 0.4994i

**Table 4:** Coordinate estimation and errors of false targets based on three transponders.

False Targets	Coordinate Estimation (M)	Error (M)
$p_{1}$	(0.4, 7969, 19.34)	(0.4, −1, −0.66)
$p_{2}$	(0, 8031, 39.82)	(0, 1, −0.18)
$p_{3}$	(0, 8060.5, 29.65)	(0, 0.5, −0.35)
$p_{4}$	(−30.5, 8060.5, 25.21)	(−0.5, 0.5,0.21)
$p_{5}$	(30.5, 8061, 24.47)	(0.5, 1, −0.53)

**Table 5:** Coordinate estimation and errors of three false point targets from the 3D deceptive template.

Jamming Type	False Targets	Coordinate Estimation (M)	Error (M)
Three transponders	$q_{1}$	(−0.48, 7973.0, 34.40)	(−0.48, 0, −0.60)
	$q_{2}$	(−15.48, 7962.6, 7.41)	(−0.48, 0.6, 0.47)
	$q_{3}$	(9.93, 7981.3, 14.01)	(−0.07, 0.3, 0.66)
Two transponders	$q_{1}$	(−0.48, 7973.0, 10.31)	(−0.48, 0, −24.69)
	$q_{2}$	(−15.48, 7962.6, 10.11)	(−0.48, 0.6, −3.06)
	$q_{3}$	(9.93, 7981.3, 6.40)	(−0.07, 0.3, −8.27)
